# Treatment of Colic

**Published:** 1879-08

**Authors:** 


Treatment of Colic.—Phares' method consists in *inversion*—that is, simply in turning the patient upside-down. Colic of several days duration has thus been relieved in a few minutes. The patient may take the elbow-knee position, or may lie (face down) on the edge of the bed, with his head and shoulders hanging down. Complete inversion, however, is best. The mechanical aid, in giving vent to gases, is, perhaps, the most efficient element in the cure.—*Jour. des Sci. Med.*

